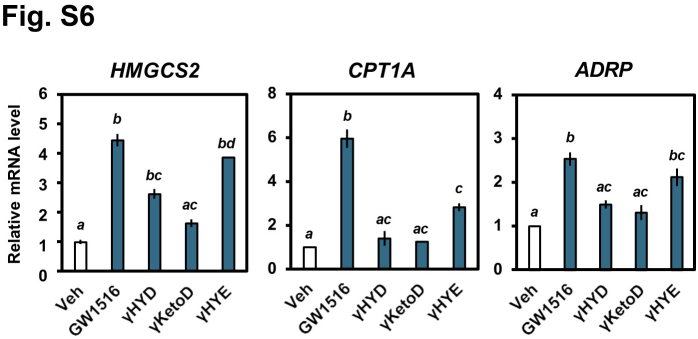# Lactic acid bacteria–derived γ-linolenic acid metabolites are PPARδ ligands that reduce lipid accumulation in human intestinal organoids

**DOI:** 10.1016/j.jbc.2022.102534

**Published:** 2022-09-24

**Authors:** Makoto Noguchi, Makoto Shimizu, Peng Lu, Yu Takahashi, Yoshio Yamauchi, Shintaro Sato, Hiroshi Kiyono, Shigenobu Kishino, Jun Ogawa, Koji Nagata, Ryuichiro Sato

**Affiliations:** 1 Nutri-Life Science Laboratory, Department of Applied Biological Chemistry, Graduate School of Agricultural and Life Sciences, The University of Tokyo, Tokyo, Japan; 2 Food Biotechnology and Structural Biology Laboratory, Department of Applied Biological Chemistry, Graduate School of Agricultural and Life Sciences, The University of Tokyo, Tokyo, Japan; 3 Food Biochemistry Laboratory, Department of Applied Biological Chemistry, Graduate School of Agricultural and Life Sciences, The University of Tokyo, Tokyo, Japan; 4 Department of Microbiology and Immunology, School of Pharmaceutical Sciences, Wakayama Medical University, Wakayama, Japan; 5 Mucosal Immunology and Allergy Therapeutics, Institute for Global Prominent Research, Future Medicine Education and Research Organization, Chiba University, Chiba, Japan; 6 Division of Applied Life Sciences, Graduate School of Agriculture, Kyoto University, Kyoto, Japan

**Keywords:** PPARδ, PPAR, nuclear hormone receptor, fatty acid, γ-linolenic acid, organoids, intestine, gut microbiota, fatty acid oxidation, lactic acid bacteria, transcription factor, ALA, α-linolenic acid, CLD, cytosolic lipid droplet, DHA, docosahexaenoic acid, GLA, γ-linolenic acid, GPCR, G protein–coupled receptor, LA, linoleic acid, LBD, ligand-binding domain, LCFA, long-chain fatty acid, NCoR, nuclear receptor corepressor, NR, Nuclear hormone receptor, PPARδ, peroxisome proliferator-activated receptor delta, PPRE, peroxisome proliferator-response element, PUFA, polyunsaturated fatty acid, SCFA, short-chain fatty acid, SRC-1, steroid receptor coactivator-l, γHYD, 13-hydroxy-*cis*-6,*cis*-9-octadecadienoic acid, γHYE, 10,13-dihydroxy-*cis*-6-octadecenoic acid, γKetoD, 13-oxo-*cis*-6,*cis*-9-octadecadienoic acid

## Abstract

Gut microbiota regulate physiological functions in various hosts, such as energy metabolism and immunity. Lactic acid bacteria, including *Lactobacillus plantarum*, have a specific polyunsaturated fatty acid saturation metabolism that generates multiple fatty acid species, such as hydroxy fatty acids, oxo fatty acids, conjugated fatty acids, and *trans*-fatty acids. How these bacterial metabolites impact host physiology is not fully understood. Here, we investigated the ligand activity of lactic acid bacteria–produced fatty acids in relation to nuclear hormone receptors expressed in the small intestine. Our reporter assays revealed two bacterial metabolites of γ-linolenic acid (GLA), 13-hydroxy-*cis*-6,*cis*-9-octadecadienoic acid (γHYD), and 13-oxo-*cis*-6,*cis*-9-octadecadienoic acid (γKetoD) activated peroxisome proliferator-activated receptor delta (PPARδ) more potently than GLA. We demonstrate that both γHYD and γKetoD bound directly to the ligand-binding domain of human PPARδ. A docking simulation indicated that four polar residues (T289, H323, H449, and Y473) of PPARδ donate hydrogen bonds to these fatty acids. Interestingly, T289 does not donate a hydrogen bond to GLA, suggesting that bacterial modification of GLA introducing hydroxy and oxo group determines ligand selectivity. In human intestinal organoids, we determined γHYD and γKetoD increased the expression of PPARδ target genes, enhanced fatty acid β-oxidation, and reduced intracellular triglyceride accumulation. These findings suggest that γHYD and γKetoD, which gut lactic acid bacteria could generate, are naturally occurring PPARδ ligands in the intestinal tract and may improve lipid metabolism in the human intestine.

Gut microbiota plays diverse critical roles in host physiological processes including energy metabolism. Dysbiosis, that is, an imbalance in microbiota, is associated with metabolic disorders such as obesity (1, 2). Human gut microbiota mainly comprises of four phyla: *Bacteroidetes*, *Firmicutes*, *Actinobacteria*, and *Proteobacteria* (3, 4). Although a relationship between the gut microbiota and metabolic diseases is not fully understood, alteration of the proportion of *Bacteroidetes* and *Firmicutes* may associate with the development of obesity (5). Previous studies provided evidence that gut microbiota alters host energy harvest. For example, fecal transplantation from *ob*/*ob* mice to germ-free mice leads to a higher increase in body fat in the WT mice (6). Gut microbiota–derived metabolites mediate host energy metabolism. For instance, changes in the amount of short-chain fatty acids (SCFAs) produced from dietary fibers have been detected in the intestinal lumen of obese patients (7, 8). SCFAs reportedly activate GPR41 and GPR43 as mediators and energy resources for host tissues (9). Activated GPR41 and GPR43 by SCFAs may function as energy sensors in gut microbiota communication connecting with the sympathetic nervous system and white adipose tissue (10, 11). In addition, long-chain fatty acids (LCFAs) may also function as nutrients and signaling molecules that target some G protein–coupled receptors (GPCRs) and are involved in systemic energy metabolism (12). However, whether LCFA derivatives converted by gut microbiota regulate host energy metabolism is unclear. In a previous study, we reported that lactic acid bacteria can enzymatically convert and produce certain fatty acids from diet-derived polyunsaturated fatty acids (PUFAs), including γ-linolenic acid (GLA) (13). *Lactobacillus acidophilus* and *Pediococcus* sp. exhibit saturation metabolism on Δ9 and Δ12 double bonds in PUFAs, producing hydroxy- and oxo-fatty acids (Fig. S1) (14). These metabolites occur at significantly higher levels in specific pathogen-free mice relative to the levels in germ-free mice (13). Although these compounds are presumed to function in the human intestine at physiological concentrations, such biological activities are yet to be studied in detail.

Nuclear hormone receptors (NRs) are transcription factors activated by lipophilic ligands, including steroid hormones, fat-soluble vitamins, and dietary lipids. The NR superfamily comprises 48 members in humans (15). An N-terminal domain characterizes these receptors with activation function 1 which can activate transcription constitutively in the absence of ligand, a central DNA-binding domain, and a C-terminal ligand-binding domain (LBD) with activation function 2 (16). The transcriptional activity of NRs is regulated by the recruitment of coactivators such as steroid receptor coactivator-l (SRC-1) and corepressors such as nuclear receptor corepressor (NCoR) (17). Ligand binding induces a conformational change of the LBD of NR and releases bound corepressors. Subsequently, coactivators are associated with and activate NRs, leading to transcriptional activation. Although the classical NRs such as steroid hormone receptors are translocated from the cytoplasm to the nucleus in a ligand-dependent manner, other NRs including peroxisome proliferator-activated receptors (PPARs) are constitutively localized in the nucleus and associated with their response elements (18). NRs govern the expression of genes involved in diverse physiological functions including reproduction, immune response, and energy metabolism. In several studies, NRs reportedly play an important role in energy metabolism. For example, NRs that regulate energy metabolism are highly expressed in metabolic tissues, including the liver, adipose tissue, skeletal muscle, and intestines (19). In the intestines, higher levels of dietary lipophilic molecules such as fatty acids, bile acids, and fat-soluble vitamins, which can serve as physiological ligands for NRs, are observed relative to the levels found in other tissues (18, 20).

PPARs play a major regulatory role in energy metabolism among the NR superfamily. They bind to the peroxisome proliferator-response element (PPRE) on the gene promoter as a heterodimer with retinoid X receptors (21). Among three PPAR subtypes [α, γ, and δ (also referred to as β)], PPARα, highly expressed in the liver, binds fatty acids or lipid-lowering fibrates (22) and controls the expression of target genes related to fatty acid β-oxidation and transport. PPARα-deficient mice develop hepatic steatosis during fasting or high-fat diet feeding. Contrastingly, PPARγ, highly expressed in the adipose tissue as a master regulator of adipogenesis, is a target molecule of the insulin sensitizer thiazolidinedione (23). PPARδ, the most recently studied PPAR subtype, is expressed in various tissues including gastrointestinal tissues as compared to PPARα and PPARγ and regulates fatty acid and glucose metabolism in metabolic tissues (19, 24, 25, 26). PPARδ activation by its specific agonist GW1516 increases expression of genes related to fatty acid β-oxidation (*e.g.*, *CPT1A* and *HMGCS2*). PPARδ also regulates cholesterol and lipoprotein metabolism in intestine (27, 28, 29). These findings show that PPARδ plays a crucial role in intestinal energy metabolism. In addition to normal physiology, a role of PPARδ on colon cancer has been reported (25, 30, 31). Although several studies showed that PPARδ is protumorigenic, protective effects of PPARδ were also reported, suggesting a complex and diverse role of PPARδ in colon cancer.

The present study explored the intestinal NRs activated by lactic acid bacteria–derived fatty acids. Using cell-based reporter assays, we found that two GLA (18:3n-3)-derived fatty acids, 13-hydroxy-*cis*-6,*cis*-9-octadecadienoic acid (γHYD) and 13-oxo-*cis*-6,*cis*-9-octadecadienoic acid (γKetoD), activate human PPARδ potently. Additionally, γHYD and γKetoD stimulated fatty acid β-oxidation in human intestinal organoids. Our findings suggest that certain fatty acids produced by gut microbiota might regulate lipid metabolism in the human intestine and improve host energy metabolism.

## Results

### Identification of intestinal NRs activated by lactic acid bacteria–derived fatty acids

**Figure 1:**
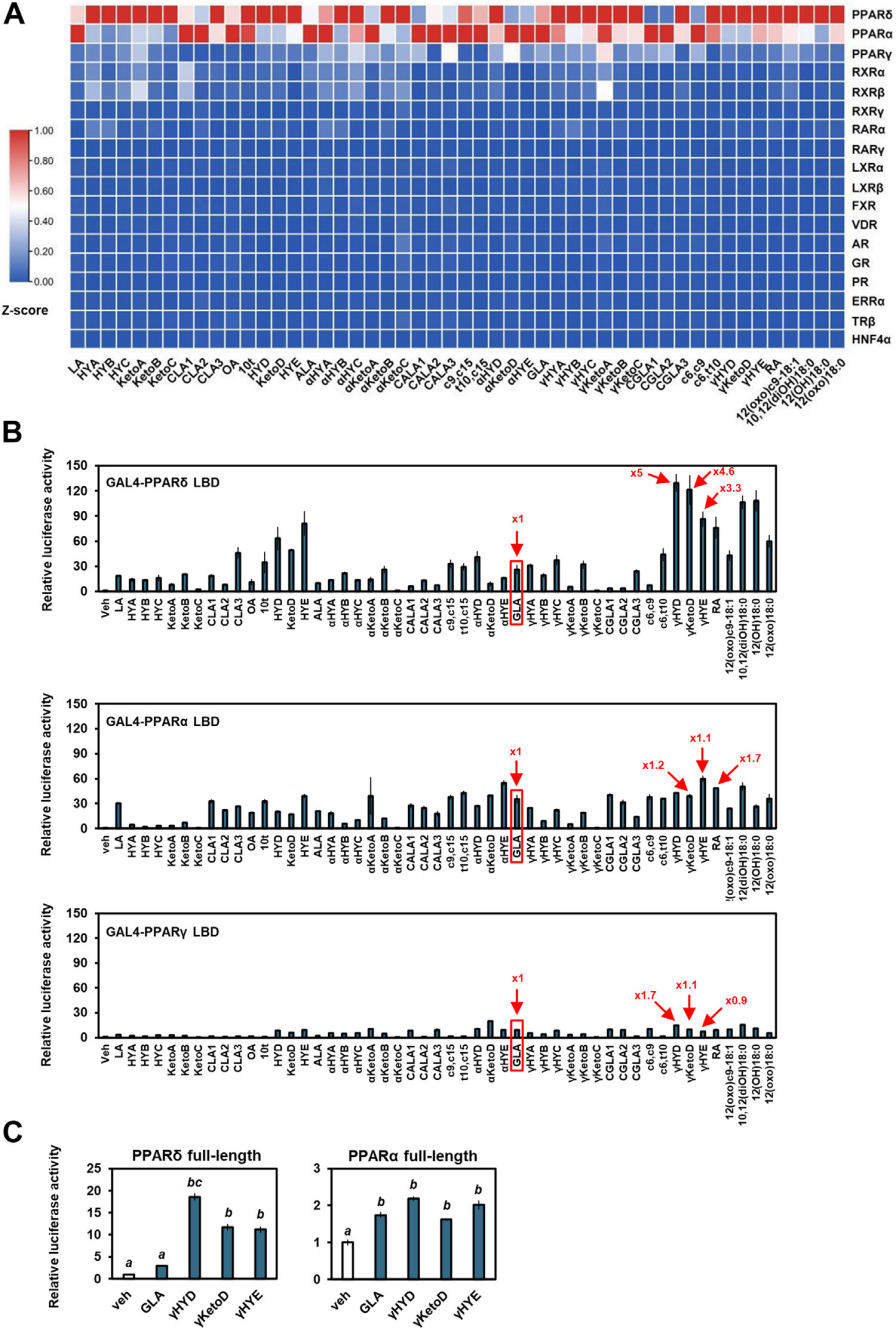
**Gut microbial γ-linolenic acid metabolites show selective activation of PPARδ and PPARα.***A* and *B*, representative data of the ligand screening of GAL4-receptor luciferase assays. HEK293 cells were cotransfected with the GAL4-NRs LBD expression plasmids and a luciferase reporter gene and treated with 100 μM fatty acids. *C*, effects of fatty acids on full-length PPARs in a luciferase reporter assay in which HEK293 cells were used. Data represent means ± SEM (n = 3), and Tukey’s test was used for analysis. Different letters indicate significant differences between groups (*p* < 0.05). Veh, vehicle; ALA, α-linolenic acid; GLA, γ-linolenic acid; LA, linoleic acid; LBD, ligand-binding domain; NR, nuclear hormone receptor; PPAR, peroxisome proliferator-activated receptor.

We performed cell-based luciferase reporter assays to identify intestinal NR activators and screened 50 fatty acids produced by gut lactic acid bacteria (13). For screening, chimeric receptors containing the LBD of various NRs that are highly expressed in the intestine (19) and the DNA-binding domain of the yeast transcription factor GAL4 (GAL4-NRs) were used (32). Luciferase reporter assays using HEK293 cells showed that lactic acid bacteria–derived fatty acids preferencely activates PPARs, especially PPARα and PPARδ (Fig. 1*A*). Other NRs were not stimulated by these fatty acids. Interestingly, some saturated metabolites of GLA, namely γHYD, γKetoD, and 10,13-dihydroxy-*cis*-6-octadecenoic acid (γHYE), had a more robust PPARδ activation than their original compound, GLA (Fig. 1*B*). These activations were not seen in PPARα, indicating a PPAR subtype-selective fatty acid sensing. Similar activation of PPARδ by saturated metabolites of linoleic acid (18:2n–6, LA) and α-linolenic acid (18:3n–3, ALA) were also observed. Because activation of PPARα and PPARγ by lactic acid bacteria–derived metabolites of LA and ALA were studied previously (33, 34, 35), our present study focused on PPARδ and GLA.

To investigate the specificity of GLA, the activation of PPARs by other 18-carbon PUFAs, that is, LA and ALA (14) was analyzed (Fig. 1*B*). Modification of LA (HYD, KetoD, and HYE) and ALA (αHYD, αKetoD, and αHYE) had little or no effect on the ligand-induced activation of PPARα. Interestingly, 13-hydroxylation of LA and ALA, as well as GLA, potently increased PPARδ activation. 13-oxo and 13-dihydroxy LA, but not ALA, also activate PPARδ. We next investigated transactivation of full-length PPARs by fatty acids. The expression plasmid for PPARs was transfected into HEK293 cells with a luciferase reporter plasmid containing PPRE, and then luciferase activity was measured. Consistent with the GAL4-NR–related results, GLA-derived fatty acids (γHYD, γKetoD, and γHYE) but not GLA activated PPARδ potently (Fig. 1*C*). In contrast, PPARα was activated almost equally by GLA and GLA-derived fatty acids. These results indicate that GLA saturation selectively activates PPARδ.

### γHYD and γKetoD are potential activators of PPARδ

**Figure 2:**
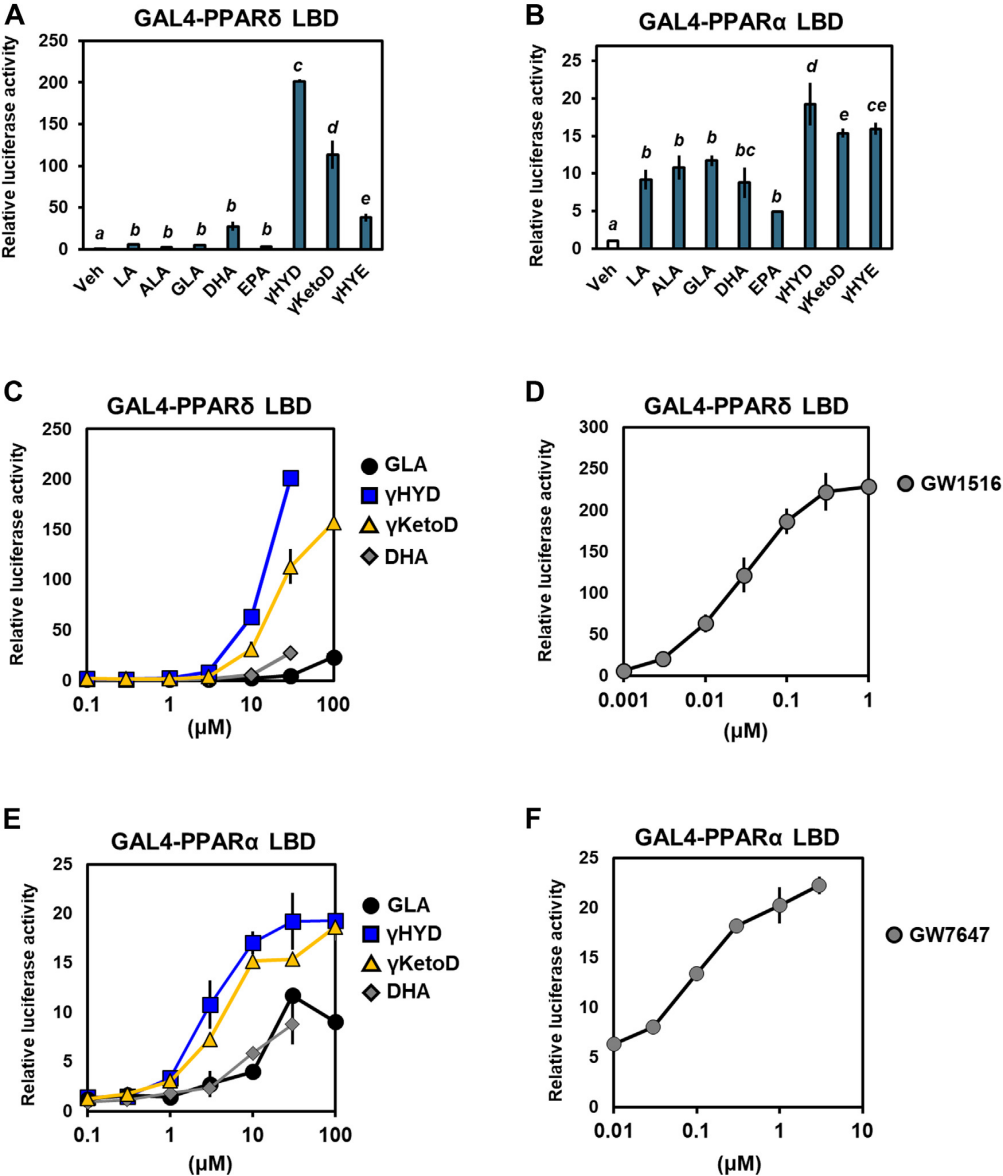
**GLA-derived fatty acids are potential naturally occurring PPARδ activators.***A* and *B*, effects of GLA metabolites and PUFAs on PPARs in GAL4-receptor luciferase assays. HEK293 cells were cotransfected with the GAL4-PPARs LBD expression plasmids and a luciferase reporter gene and treated with 30 μM fatty acids. *C*–*F*, comparative dose-response relationships of PPARδ (*C* and *D*) and PPARα (*E* and *F*). EC_50_s were calculated using the results in (*C*–*F*), as shown in Table 1. Data represent means ± SEM (n = 3), and Tukey’s test was used for analysis. Different letters indicate significant differences between groups (*p* < 0.05). PUFA, polyunsaturated fatty acid; PPAR, peroxisome proliferator-activated receptor; GLA, γ-linolenic acid; LBD, ligand-binding domain.

We also examined the activation of PPARs by other dietary PUFAs, namely docosahexaenoic acid (22:6n–3, DHA) and eicosapentaenoic acid (20:5n–3), which have been reported as natural occurring ligands for PPARs (36, 37). GLA-derived fatty acids, especially γHYD and γKetoD, activated PPARδ more robustly than DHA and eicosapentaenoic acid as well as GLA, whereas the activation of PPARα by these PUFAs was similar (Fig. 2, *A* and *B*); thus, GLA-derived fatty acids are potential naturally occurring activators of PPARδ. In a luciferase reporter assay in which GAL4-PPARs were used, PPAR activations by fatty acids and PPAR agonists were dose-dependent (Fig. 2, *C–F*). As matched with Figure 1, PPARδ activation by γHYD and γKetoD was potent [effective concentration for half-maximal response (EC_50_) = 10.6 and 20.3 μM, respectively, Table 1]. These activation efficacies were much higher than that of GLA (EC_50_ = 509 μM), indicating that γHYD and γKetoD are potential activators of PPARδ.

**Table 1:** EC50 values of GLA-derived fatty acids in relation to PPARδ (*A*) and PPARα (*B*)

Ligand/fatty acids	EC_50_ (μM)
A. GAL4-PPARδ LBD	
GW1516	0.025
DHA	44.559
GLA	509.000
γHYD	10.585
γKetoD	20.324
B. GAL4-PPARα LBD	
GW7647	0.106
DHA	9.324
GLA	11.003
γHYD	2.842
γKetoD	4.136

Values were calculated from luciferase activity using a Quest Graph EC50 Calculator.

### GLA-derived fatty acids regulate the recruitment of coactivators and corepressors

**Figure 3:**
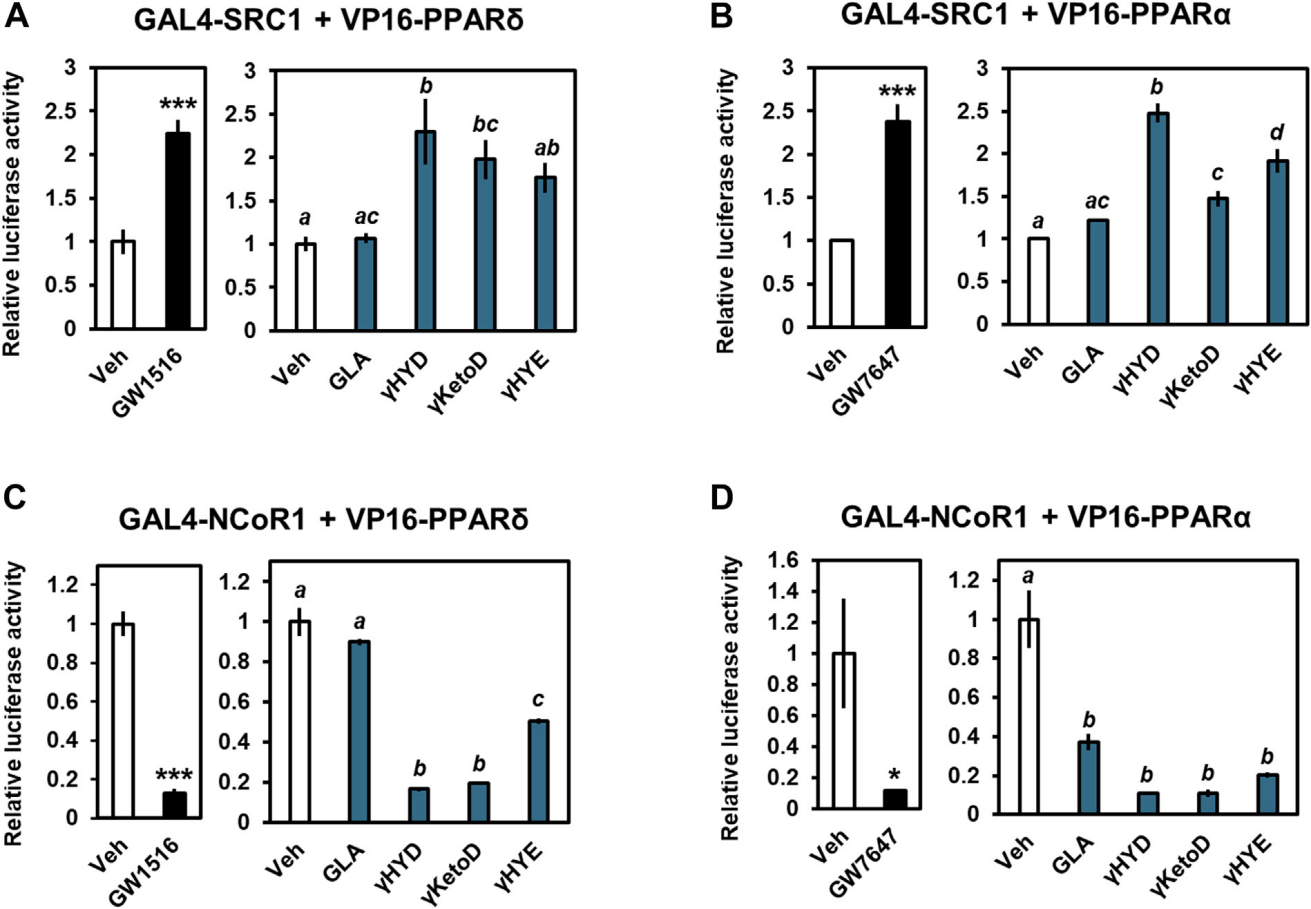
**GLA-derived fatty acids regulate coactivator/corepressor exchange.***A* and *B*, mammalian two-hybrid assay of PPARs and the coactivator SRC-1. HEK293 cells were cotransfected with VP16-PPARδ (*A*) or VP16-PPARα (*B*). *C* and *D*, mammalian two-hybrid assay of PPARs and the corepressor NCoR. HEK293 cells were cotransfected with VP16-PPARδ (*C*) or VP16-PPARα (*D*). The ligands used were 100 nM GW1516 (PPARδ agonist), 1 μM GW7647 (PPARα agonist), or 30 μM fatty acids. Data represent means ± SEM (n = 3), and Student’s *t* test or Tukey’s test was used for analysis. ∗*p* < 0.05 and ∗∗∗*p* < 0.001 or different letters indicate significant differences between groups (*p* < 0.05). Veh, vehicle; GLA, γ-linolenic acid; NCoR, nuclear receptor corepressor; SRC-1, steroid receptor coactivator-1; PPAR, peroxisome proliferator-activated receptor.

We performed mammalian two-hybrid assays to determine the mechanism underlying the activation of PPARs by GLA derivatives. NRs, including PPARs, interact with corepressors, such as NCoR without ligands. Upon ligand binding, NRs release the corepressors and recruit coactivators such as SRC-1, inducing transcription (17). Expression plasmids for GAL4-SRC-1 contain GAL4 and the receptor-interacting domain of the coactivator SRC-1 and VP16-PPARs, which contain PPARs and the transactivation domain of the herpes virus VP16 protein and were used. As reported previously, PPAR activation by specific agonists increased the ligand-dependent recruitment of SRC-1 to PPARs (Fig. 3, *A* and *B*). Additionally, γHYD, γKetoD, and γHYE but not GLA induced coactivator-recruitment to PPARs. Importantly, these data are consistent with the activation of PPARs (Fig. 1*B*). Similar to the SRC-1–related results, γHYD, γKetoD, and γHYE reduced the interaction between PPARs and corepressor NCoR (Fig. 3, *C* and *D*). Thus, GLA-derived fatty acids activate PPARs through the association/dissociation of coactivators and corepressors.

### GLA-derived fatty acids bind directly to human PPARδ

**Figure 4:**
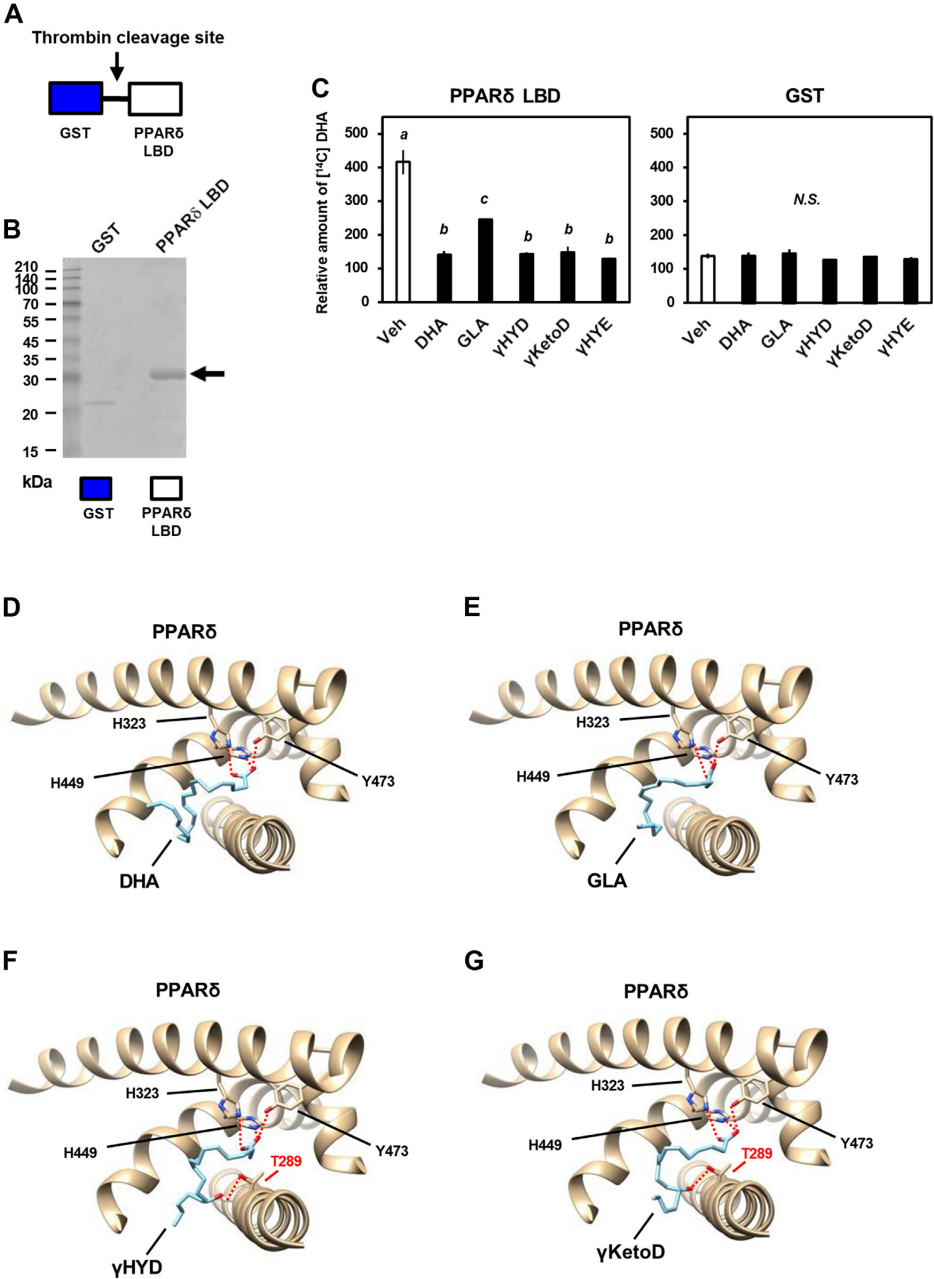
**Binding of GLA-derived fatty acids to PPARδ.***A*, a construction of GST-human PPARδ LBD protein. *B*, purified human PPARδ LBD recombinant protein used in the binding assay was analyzed using Coomassie Brilliant Blue staining. *C*, competitive binding of GLA-derived fatty acids to purified human PPARδ LBD proteins in the presence of 100 nM [^14^C] DHA and 1 μM nonlabeled fatty acids. (*D*–*G*), binding model of DHA (*D*), GLA (*E*), γHYD (*F*), and γKetoD (*G*) to human PPARδ (PDB: 3gwxA), as analyzed using AutoDock Vina. Data represent means ± SEM (n = 3), and Tukey’s test was used for analysis. Different letters indicate significant differences between groups (*p* < 0.05). CBB, Coomassie Brilliant Blue; PPAR, peroxisome proliferator-activated receptor; GLA, γ-linolenic acid; DHA, docosahexaenoic acid; LBD, ligand-binding domain; γHYD, 13-hydroxy-*cis*-6,*cis*-9-octadecadienoic acid; γKetoD, 13-oxo-*cis*-6,*cis*-9-octadecadienoic acid.

To identify the mechanism underlying the activation of PPARδ by GLA-derived fatty acids, we performed a [^14^C] DHA competitive ligand-binding assay describing the displacement of ligands from receptors with recombinant human PPARδ LBD protein produced by a bacterial expression system (Figs. 4, *A* and *B* and S2). PPARδ LBD or glutathione-S-transferase (GST) recombinant protein was incubated with both [^14^C] DHA and nonradioactive competitors including GLA derivatives. A competitive inhibition of [^14^C] DHA binding was analyzed as a direct binding to PPARδ LBD (see Experimental procedures). Similar to the DHA-related results, GLA derivatives (γHYD, γKetoD, and γHYE) displaced [^14^C] DHA binding to PPARδ LBD competitively (Fig. 4*C*). Consistent with transactivation, the only weak competition was observed with GLA, which activates PPARs moderately, and no competition was observed with GST protein. These results suggest that GLA-derived fatty acids bind directly to human PPARδ.

Next, we performed a docking simulation using AutoDock Vina (38) to construct binding models of human PPARδ LBD with GLA-derived fatty acids. Consistent with a previous study (39), human PPARδ bound to a ligand (DHA) through hydrogen bonds with polar residues (H323, H449, and Y473) (Fig. 4*D*). Furthermore, the docking results showed that both GLA and GLA-derived fatty acids also associate with these polar residues (Fig. 4, *E–G*). Interestingly, the additional polar residue (T289) formed hydrogen bonds selectively with GLA-derived fatty acids but not GLA (Fig. 4, *F* and *G*). H323, H449, and Y473 were associated with the carboxy-terminus of fatty acids, whereas T289 donated a hydrogen bond to C13 of γHYD or γKetoD. T289 of human PPARδ is conserved among mammals and this threonine residue is replaced by serine in PPARα and PPARγ (Fig. S3), the activations of which were not affected by the saturation of GLA. These results suggest that saturation at C13 of GLA by lactic acid bacteria may serve to donate hydrogen bonds to T289 of PPARδ and stabilize receptor-ligand docking, in a subtype-selective manner.

### Human intestinal PPARδ is activated by GLA metabolites

**Figure 5:**
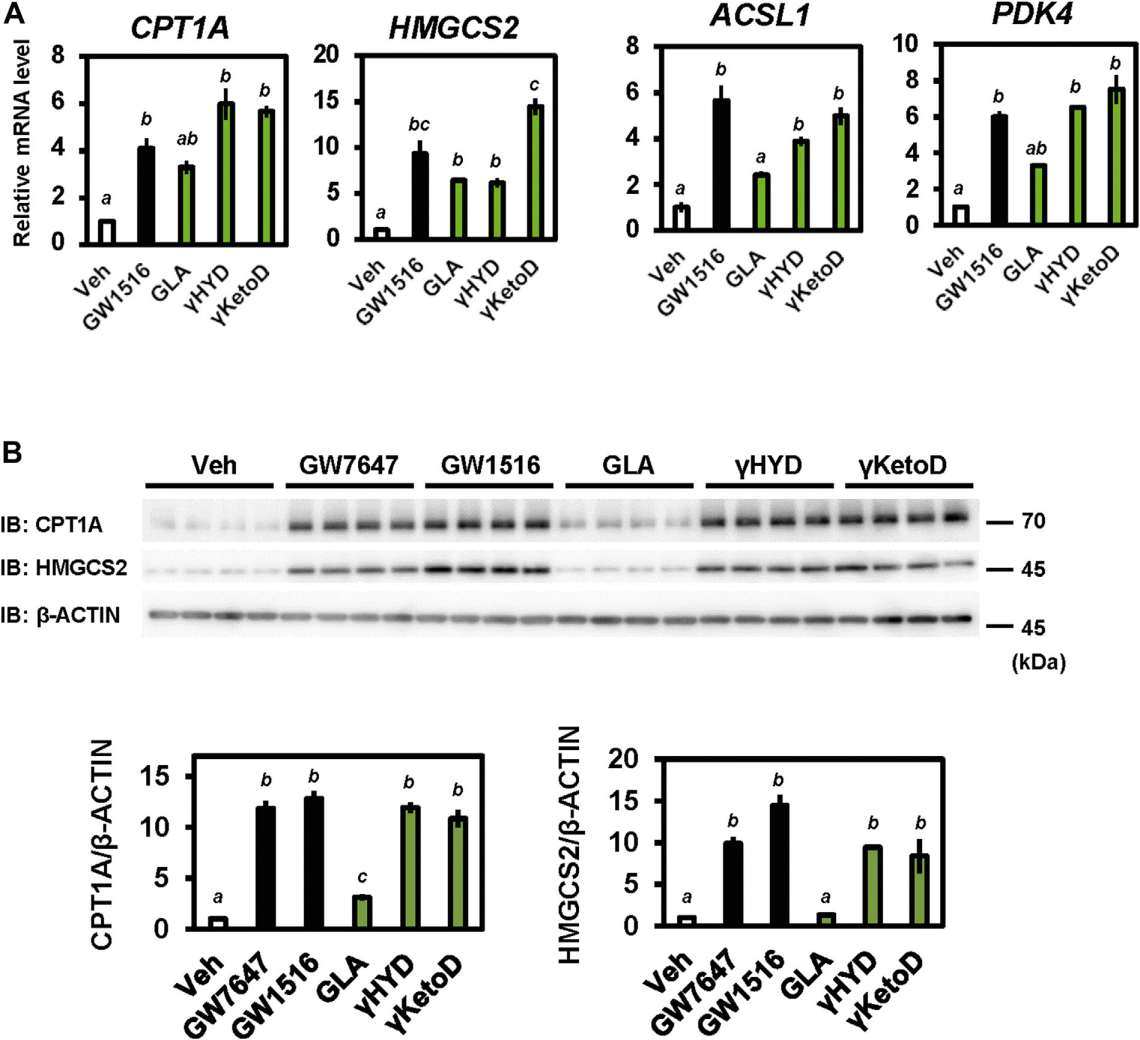
**GLA-derived fatty acids increase the expression of PPARδ target genes in human intestinal organoids.***A* and *B*, human intestinal organoids were treated with 1 μM GW1516 (PPARδ agonist) or 30 μM GLA-derived fatty acids for 24 h. Relative mRNA levels were determined using RT-qPCR (*A*). CPT1A, HMGCS2, and β-ACTIN protein levels were measured using Western blotting (*B*). Data represent means ± SEM (n = 3 or 4), and Tukey’s test was used for analysis. Different letters indicate significant differences between groups (*p* < 0.05). IB, immunoblotting; Veh, vehicle. PPAR, peroxisome proliferator-activated receptor; CPT1A, carnitine palmitoyltransferase 1A; HMGCS2, 3-hydroxy-3-methylglutaryl-CoA synthase 2; ACSL1, Acyl-CoA Synthetase Long Chain Family Member 1; PDK4, pyruvate dehydrogenase kinase 4; GLA, γ-linolenic acid.

In studies of human intestinal biology, human intestinal epithelial organoids are considered powerful tools because they represent structure and functions *in vivo*. To investigate the physiological role of GLA-derived fatty acids in human tissues, we used human-induced pluripotent stem cell–derived intestinal organoids (40). First, we confirmed that endogenous PPARδ gene expression was indistinguishable between human intestinal organoids and differentiated Caco-2 cells, that is, the human cell line which expresses endogenous PPARδ (Fig. S4*A*) (41, 42). In addition, PPARδ protein levels in human intestinal organoids are comparable to mouse tissues where PPARδ is highly expressed (Fig. S4*B*). Treatment with the PPARδ agonist GW1516 upregulated the mRNA expression of target genes in the organoids (*i.e.*, *CPT1A*, *HMGCS2*, *PDK4*, and *ASCL1*; Fig. 5*A*). In agreement with the GW1516-based results, the expression of PPARδ target genes was increased significantly by treatment with GLA-derived fatty acids relative to treatment with GLA (Fig. 5*A*). Similar results occurred when primary intestine tissue–derived human organoids were used (Fig. S4*C*). In addition, protein expression levels of CPT1A and HMGCS2 were significantly increased (Fig. 5*B*). These findings indicate that GLA-derived fatty acids activate PPARδ in human intestinal epithelial cells.

### GLA-derived fatty acids reduce lipid accumulation in human intestinal organoids

**Figure 6:**
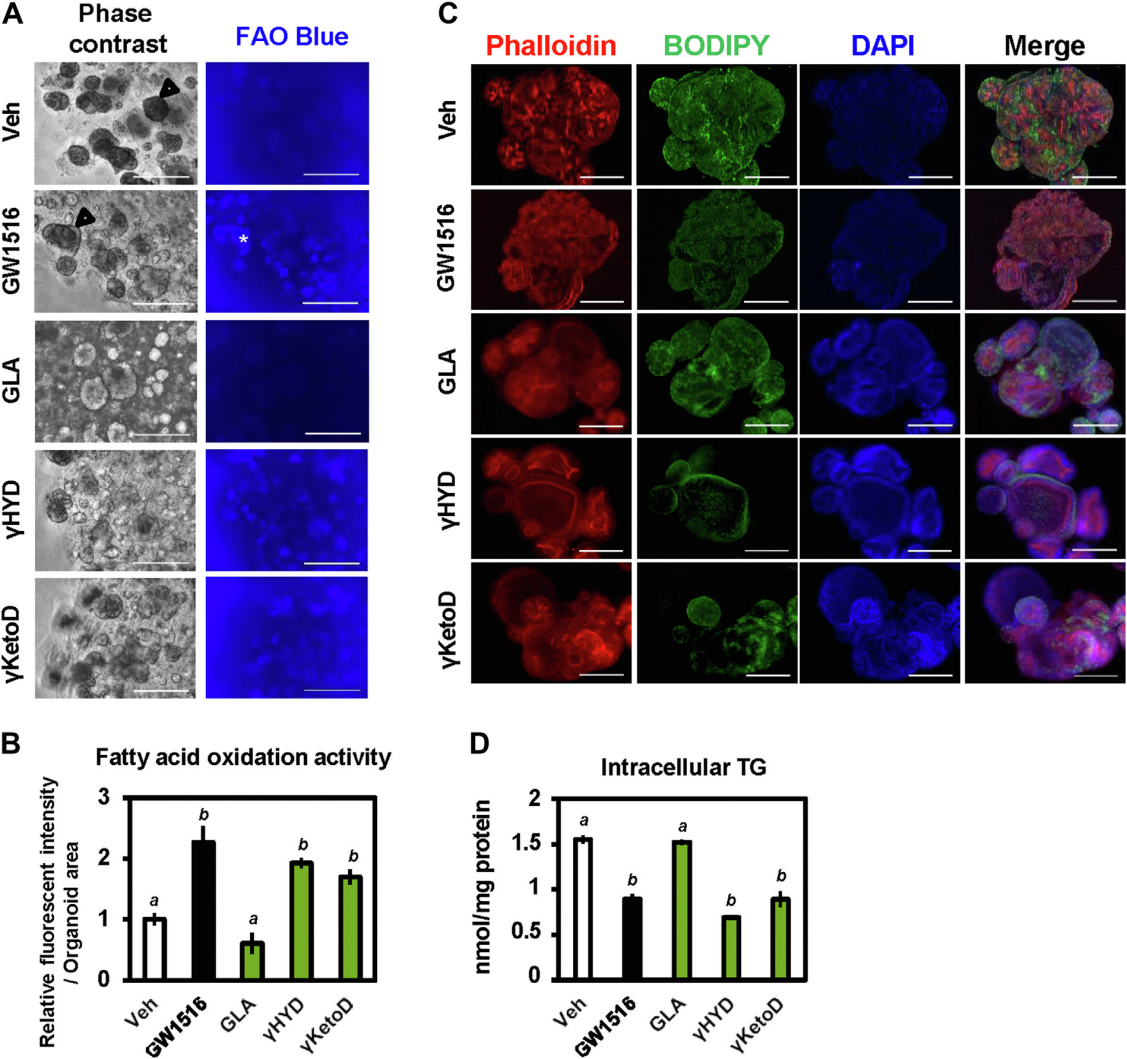
**GLA-derived fatty acids promote fatty acid β-oxidation and reduce lipid accumulation in human intestinal organoids.***A* and *B*, fluorescence detection of fatty acid β-oxidation activity in living organoids using FAOBule. Organoids were incubated with 30 μM FAOBlue at 37 °C for 30 min. The scale bar represents 500 μm (*A*). Relative fluorescence intensities were determined and normalized to the organoid surface area (*B*). *C*, triglyceride accumulation in human intestinal organoids was analyzed using BODIPY staining. Organoids were stained with Phalloidin (*red*: F-actin), BODIPY 493/503 (*green*: lipid droplets), and DAPI (*blue*: nucleus). The scale bar represents 200 μm. *D*, quantification of intracellular triglyceride. Organoids pretreated with 500 μM of oleic acid for 48 h were then treated with 1 μM GW1516 (PPARδ agonist) and 30 μM GLA-derived fatty acids for 24 h. Fluorescence imaging and fluorescence intensity were analyzed using a BZ-X800 analyzer (Keyence). Data are means ± SEM (n = 3), and Tukey’s test was used for analysis. Different letters indicate significant differences between groups (*p* < 0.05). PPAR, peroxisome proliferator-activated receptor; GLA, γ-linolenic acid.

Because activation of PPARδ leads to fat burning through induction of fatty acid β-oxidation–related target genes, we examined the regulation of lipid metabolism by GLA derivatives in human intestinal organoids. Activation of fatty acid β-oxidation in living cells was detected using the fluorescence dye FAOBlue (43). Consistent with the observed increase in mRNA levels, the PPARδ agonist and GLA-derived fatty acids but not GLA significantly increased the activity of fatty acid β-oxidation (Fig. 6, *A* and *B*). Next, we analyzed intracellular triglyceride accumulation using BODIPY staining of neutral lipid. Consistent with the activities of fatty acid β-oxidation, intracellular triglyceride levels were reduced in the presence of GLA-derived fatty acids (Fig. 6*C*). Quantitative analysis revealed that GLA-derived fatty acids but not GLA significantly reduced intracellular triglyceride levels and the PPARδ agonist (Fig. 6*D*). The PPARδ agonist and GLA-derived fatty acids do not affect cell viability (Fig. S5). Thus, GLA-derived fatty acids may reduce lipid accumulation through activation of fatty acid β-oxidation in human intestinal epithelial cells.

## Discussion

Human gut microbiota produces numerous molecules through the metabolism of dietary components, such as dietary fibers, bile acids, and amino acids. These microbiota-derived metabolites play crucial roles in regulating host metabolism (44, 45). SCFAs, one of the major microbiota-derived metabolites, are produced *via* the fermentation of dietary fibers and are the most well-studied metabolites in host–microbiota interactions by which host energy metabolism is regulated through the activation of SCFA-sensing GPCRs (*e.g.*, GPR41 and GPR43) (8, 10). Some bacteria metabolize LCFAs, especially PUFAs, to generate hydroxy fatty acids, oxo fatty acids, conjugated fatty acids, and partially saturated *trans*-fatty acids (13, 14). The biological signal of LCFAs is mainly mediated through membrane LCFA-sensing GPCRs (*e.g.*, GPR40 and GPR120) and nuclear fatty acid receptor PPARs. Previous studies showed that microbiota-derived PUFAs stimulate GPCRs and PPARs (33, 35, 46, 47, 48). Although intestinal GPCRs for microbiota-derived LCFAs in the intestine have been reported, nuclear mediators have yet to be identified. Fatty acids and fatty acid–derived molecules such as eicosanoids and endocannabinoids regulate the activities of PPARs and other NRs including retinoid X receptor, hepatocyte nuclear factor-4α, retinoic acid receptor, and liver X receptor (49, 50, 51). These NRs are highly expressed in the intestine (19), suggesting they are potential mediators of microbiota-derived fatty acid derivatives. The present study showed that GLA and GLA metabolites activate PPARs, especially PPARα and PPARδ, selectively. Fatty acids activate all PPARs as dietary ligands. Endogenous ligands for PPARα (1-palmitoyl-2-oleoyl-*sn*-glycerol-3-phosphocholine) and PPARγ (15-deoxy-Δ^12,14^-prostaglandin J_2_) have been reported (36, 52, 53, 54); prostacyclin and carbaprostacyclin were reported as endogenous ligands for PPARδ (55, 56), although they also activated other PPARs. Our study found that GLA metabolites activate PPARδ potently and to a greater extent than that achieved by other fatty acids. Lactic acid bacteria–derived fatty acids are present in the intestine, the primary site of fatty acid absorption. Although bacterial fatty acid metabolites exists lower levels than original compounds, these metabolites produced by lactic acid bacteria were detected in the intestine (13, 46), implying that substantial levels of these metabolites are present in host tissues. These findings suggest that lactic acid bacteria–derived GLA metabolites function as ligands for PPARδ.

Similar to a previous study (57), our data showed that GLA preferentially activates PPARα and PPARδ rather than PPARγ. Consistent with another study (39), our docking simulation indicated that γHYD and γKetoD are capable of binding to human PPARδ through hydrogen bonds between the fatty acids and the polar residues of human PPARδ (His323, His449, and Tyr473), which are located in arm I of the LBD. The binding ability of fatty acids to PPARδ is generally proportional to the length of their hydrocarbon chains, indicating the strength of hydrophobic interactions (57). However, in the current study, γHYD and γKetoD, both C18 fatty acids, showed comparable ligand-binding abilities to that of the C22 fatty acid DHA (Fig. 4*B*). Our docking model showed that γHYD and γKetoD but not GLA formed additional hydrogen bonds with Thr289. GLA saturation selectively activates PPARδ transactivation but not other PPARs (Fig. 1*B*). Saturations of LA (HYD, KetoD, and HYE) and ALA (αHYD) are also effective (Fig. 1*B*), suggesting that fatty acid saturation may be a common mechanism underlying the PPARδ activation. His323, His449, and Tyr473 of human PPARδ were important residues in hydrogen bonding with other fatty acids (39). These amino acid residues and Thr289 are located in arm I of the LBD, indicating the helix 12, which contributes to the ligand-dependent recruitment of the coactivator complex. Although the LBD of PPARs share 60%–70% sequence identity (58), Thr289 of human PPARδ is not conserved and replaced with the serine residue in PPARα and PPARγ (Fig. S3). Threonine and serine are similar amino acids with polar uncharged side chains, and the serine residues of PPARα (Ser280) and PPARγ (Ser289) are reported to contribute to bind other fatty acids (39). Because the ligand-binding assays of PPARα and PPARγ was not performed, further studies are required to determine how the threonine residue selectively contributes to the binding and the activation of PPARδ by GLA metabolites. In this study, the mammalian two-hybrid assay using GAL4-coactivator/corepressor and VP16-NRs was used to detect a recruitment of coactivator/corepressor to NRs in response to ligands or fatty acids. Because this model relies on the VP16 transactivation domain of a yeast protein for activity, further experiments including chromatin immunoprecipitation assay should be required to verify an endogenous interaction between them.

PPARδ is reported to play an important role as a regulator in lipid metabolism in multiple tissues, including the liver and skeletal muscle, through the regulation of fatty acid β-oxidation–related gene expression. In addition, intestinal PPARδ protects against diet-induced obesity and dyslipidemia (27). Cardiovascular disease is a major cause of death in worldwide, and postprandial hyperlipidemia is an important risk factor for cardiovascular disease (59). An absorbed dietary lipid is assembled into a chylomicron in the intestine for secretion into the lymph system in the postprandial state. Excess dietary lipids are temporally stored as cytosolic lipid droplets (CLDs) (60). In obese mice, intestinal CLDs are enlarged. They contain the distinctive expression profile of CLD-localized proteins, suggesting that the changes mentioned above prevent absorbed lipids from being mobilized and utilized effectively (61, 62, 63). In clinical and experimental studies, PUFA intake improves postprandial hyperlipidemia (64, 65). In the present study, GLA metabolites reduced triglyceride accumulation in human intestinal epithelial cells by activating fatty acid β-oxidation. Lipid transport by fatty acid transport proteins (FABP1 and FABP2) is critical for fatty acid catabolism in enterocytes. FABP1 is involved in fatty acid oxidation in the intestine, whereas FABP2 directs fatty acids to triacylglyceride synthesis (66). Consistent with these studies, we found that the expression of FABP1 but not FABP2 was increased by GLA metabolites (Fig. S4*D*), suggesting that these metabolites direct fatty acids to degradation in the intestine. Interestingly, GLA metabolites also increased the expression of PPARδ target genes in human hepatocytes (Fig. S6), indicating improved lipid metabolism in multiple tissues through PPARδ activation.

Organoids are recognized as *in vitro* organ models with highly physiological characteristics (67). Human intestinal cell lines, such as Caco-2 cells, lack some important pathways, including lipoprotein metabolism, observed *in vivo* (68, 69). In addition, PPARs exhibit marked species differences in response to ligand activation (70, 71). We found that GLA-derived fatty acids activate PPARδ and reduce lipid accumulation in human intestinal organoids. Considering these findings with evidence that microbiota-derived metabolites are present in the intestine, we suggest that GLA metabolites could improve lipid metabolism *via* PPARδ activation in the human intestine. Although intestinal organoids possess high physiological properties, *in vivo* study may need to describe whether GLA metabolites are *bona fide* physiological ligands for PPARδ.

**Figure 7:**
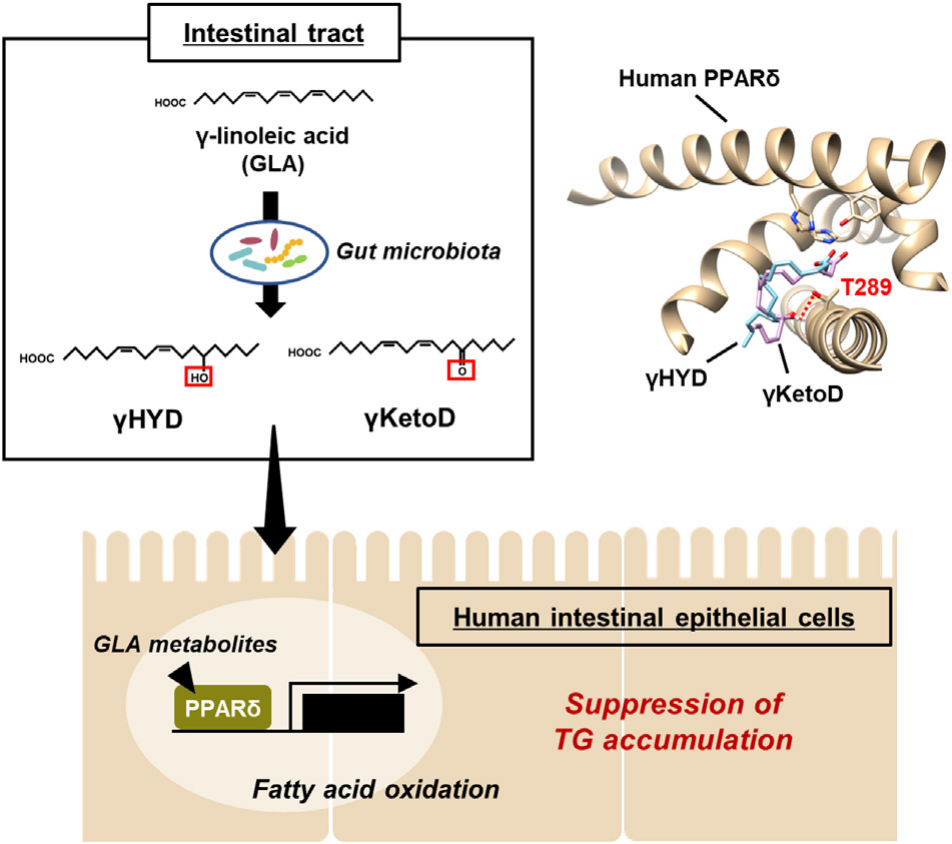
**Models of the mechanism by which lactic acid bacteria–derived GLA metabolites activate PPARδ and modulate lipid metabolism in human intestine.** GLA, γ-linolenic acid; PPARδ, peroxisome proliferator-activated receptor delta.

In conclusion, the GLA-derived fatty acids, γHYD and γKetoD, produced by lactic acid bacteria are ligands for PPARδ. GLA saturation selectively enhances PPARδ transactivation through an additional hydrogen bond between Thr289 and fatty acids. Moreover, the GLA-derived fatty acids reduce CLD levels in human intestinal epithelial cells (Fig. 7). These findings will likely contribute to the development of effective preventative treatments for metabolic disorders, including obesity, because the aforementioned fatty acid metabolites exhibited high bioactivity in reducing lipid storage in human intestinal epithelial cells.

## Experimental procedures

### Materials

**Fig.S1:**
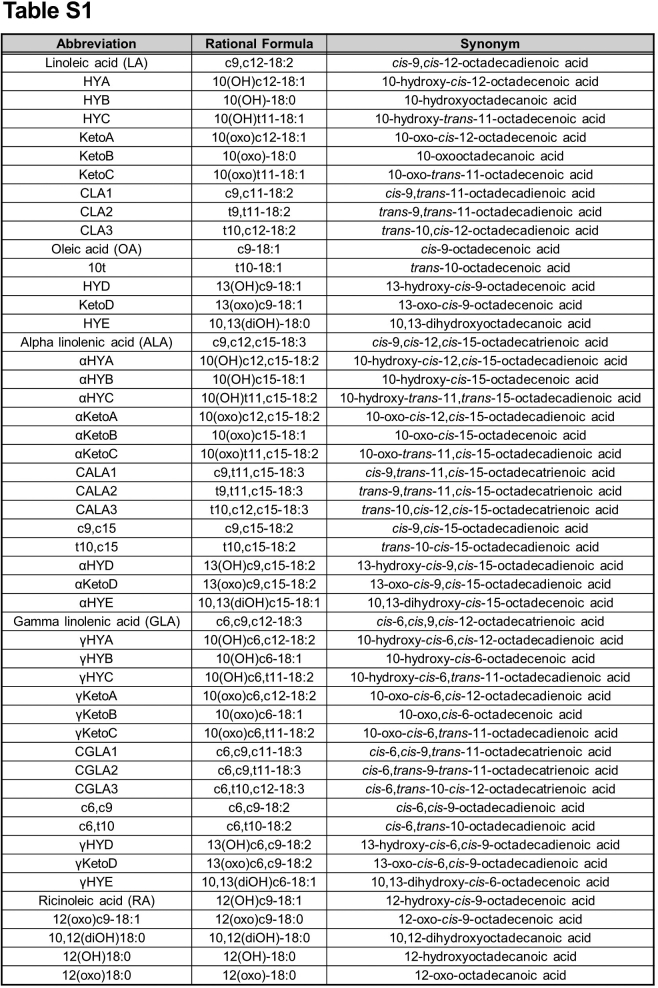


**Fig.S2:**
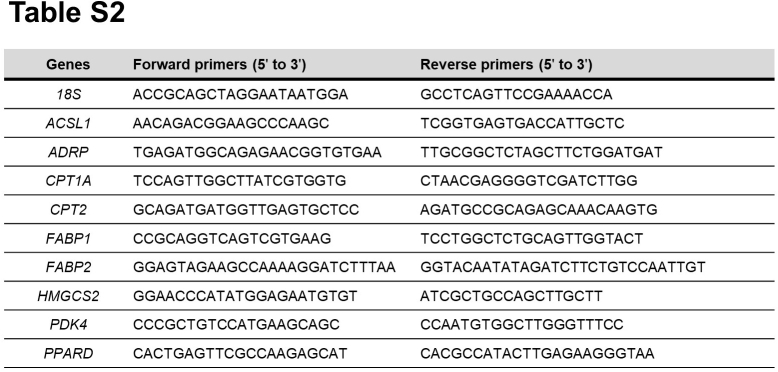


**Fig.S3:**
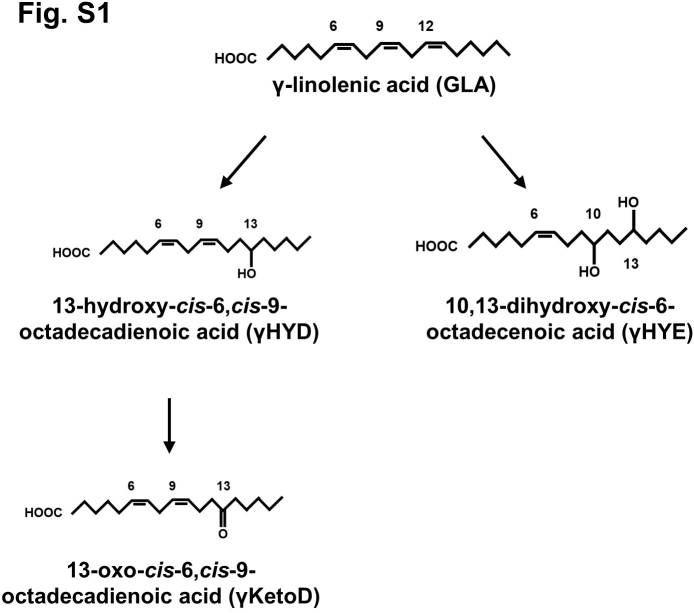


**Fig.S4:**
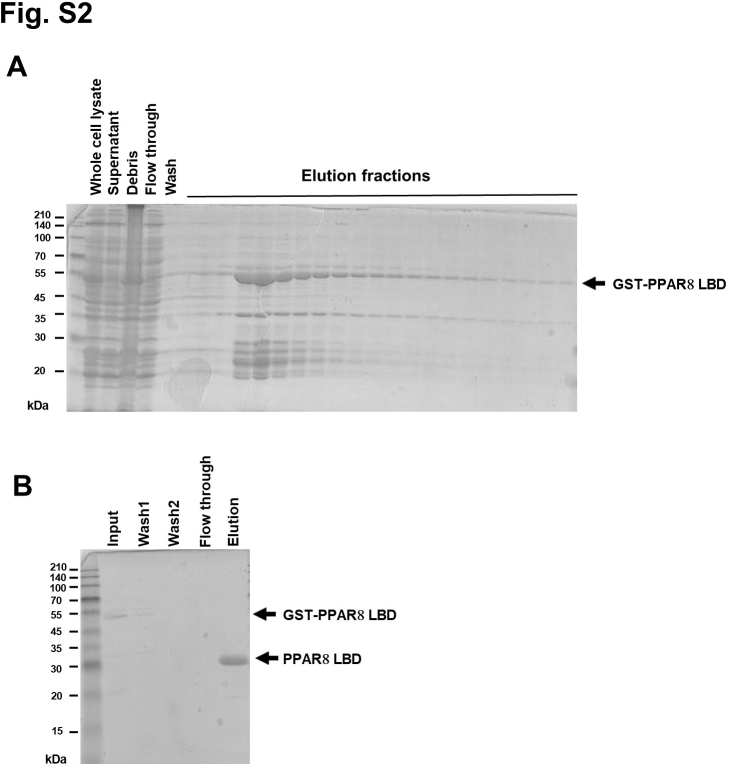


**Fig.S5:**
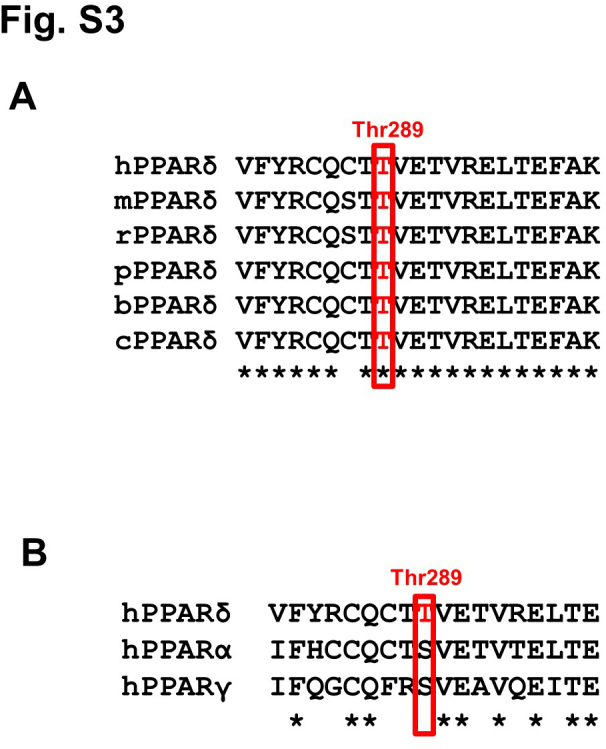


**Fig.S6:**
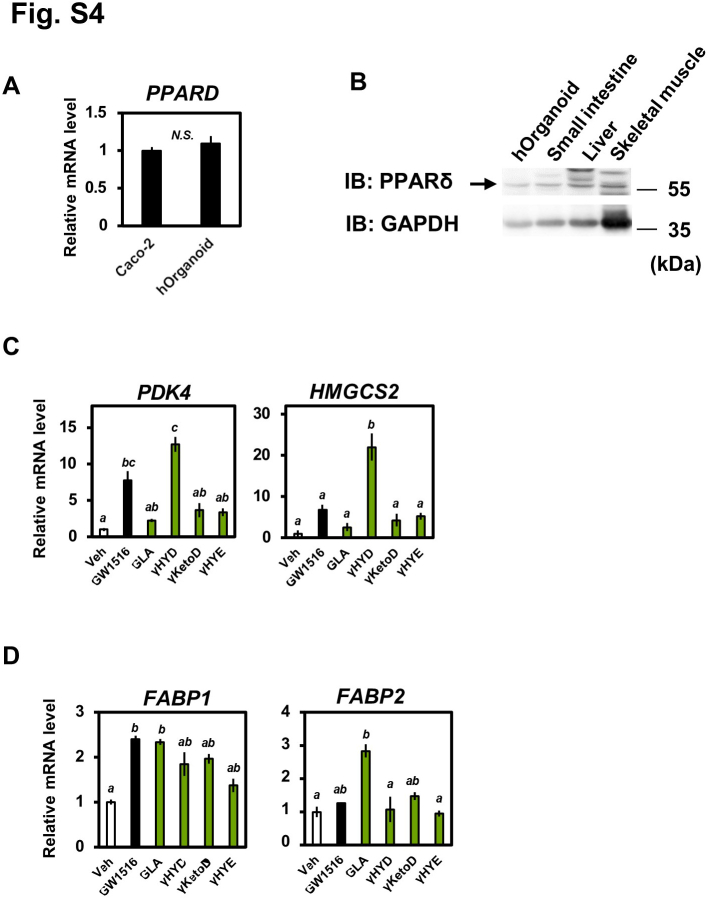


Lactic acid bacteria–derived fatty acids were produced as described previously (Fig. S1 and Table S1) (13). GW7647 and GW1516 were purchased from Sigma–Aldrich. The following antibodies were purchased from commercial sources: anti-HMGCS2 and anti-CPT1A (ab137043 and ab128568); anti-β-actin (A5441). All other chemicals of analytical grade were purchased from Sigma–Aldrich, FUJIFILM Wako Pure Chemical, or nakalai tesque.

### Animals

C57BL/6J mice were purchased from Nihon Crea. Mice were housed with a 12:12-h light–dark cycle and provided free access to water and standard diet (Labo MR Stock; Nosan Corporation Bio-Department). All animal experiments were conducted according to the guidelines of the Animal Usage Committee of the University of Tokyo. The experiments using animal studies were approved by the Animal Usage Committee of The University of Tokyo.

### Cell culture

HEK293 cells were maintained in Dulbecco’s modified Eagle’s medium (DMEM) supplemented with 10% fetal bovine serum, 100 units/ml penicillin, and 100 μg/ml streptomycin. As described previously, human intestinal organoid cultures and passages were conducted (40). Briefly, organoids were cultured with a 25% L-WRNH–conditioned medium embedded in Matrigel Matrix (Corning). The entire medium was changed every 2 to 3 days, and passages were performed every 5 to 6 days. Following passages, cell dissociation was performed using TrypLE express (Corning) at a general ratio of 1:8 embedded in Matrigel Matrix on a Nunclon Delta 4-well dish (Thermo Scientific). All cells were cultured at 37 °C in a 5% CO_2_ atmosphere. The experiments using primary human organoids complied with the Declaration of Helsinki and were approved by the human ethical committee of The University of Tokyo and Osaka University. All tissues were sampled with informed consent.

### Cotransfection and luciferase assay

HEK293 cells were plated in 96-well plates (Greiner Bio-One) and cultured on DMEM medium supplemented with 10% charcoal-treated fetal bovine serum, 100 units/ml penicillin, and 100 μg/ml streptomycin for 24 h. After that, they were transfected with 50 ng of one of the reporter plasmids, 40 ng of one of each of the expression plasmids, and 10 ng of pCMV-β-Gal (an expression plasmid for β-galactosidase) using the calcium phosphate method. At 24 h after transfection, vehicle, fatty acids, or ligands were added. After incubation for an additional 24 h, luciferase activity was measured using a SpectraMax M2 (Molecular Devices). Luciferase activities were normalized to β-galactosidase activities. Plasmids were generous gifts from Dr Makoto Makishima (GAL4-SRC1 and GAL4-NCoR), Dr David Mangelsdorf and Dr Steven Kliewer (pCMX, pCMX-GAL4-NR LBD, TK-MH100×4-luc, pCMX TK-PPREx3-luc, and TK-luc), and Dr Kazuhiko Umesono (pCMX-PPARδ).

### RNA isolation and real-time quantitative PCR

Total RNA was extracted from cells or organoids using ISOGEN (Nippon gene) per the manufacturer’s instructions. cDNA was synthesized and amplified from total RNA using a High-Capacity cDNA Reverse Transcription Kits (Applied Biosystems). Real-time quantitative PCR was performed using an Applied Biosystems StepOnePlus Real-Time PCR System (Thermo Fisher Scientific) and FastStart Universal SYBR Green Master (Roche Applied Science) per the manufacturer's protocols. All relative mRNA expression levels were normalized to that of *18S*. Primer sequences are listed in Table S2.

### Western blot analysis

Cells or organoids were treated as described in the figure legends. After treatment, protein extraction was prepared using a cell suspension in RIPA buffer [50 mM Tris‒HCl (pH 8.0), 1 mM EDTA, 150 mM NaCl, 1% NP-40, and 0.25% sodium deoxycholate] supplemented with protease inhibitors. All samples were treated with 6× Laemmli buffer [1 mM Tris‒HCl (pH 6.8), 30% glycerol, 10% SDS, 600 mM DTT, and 0.03% bromophenol blue]. The protein samples were analyzed by SDS-PAGE followed by immunoblotting with the indicated antibodies and visualized by Amersham Bioscience ECL Western Blotting Detection Reagent (GE Healthcare Life Sciences) or Immobilon Western Chemiluminescent HRP Substrate (Merck Millipore).

### Recombinant protein expression and purification

Human PPARδ LBD (140–442) construct was cloned into a pGEX-4T1 vector (GE Healthcare Life Sciences) at the restriction enzyme site of *Sma*I and *Xho*I. The protein was expressed in *Eschericia coli* BL21 (DE3) pLys cells (Novagen) as a C-terminal fusion to a GST tag with a thrombin cleavage site. A single colony was cultured in 2 ml of LB media supplemented with 20 μg/ml of ampicillin and 30 μg/ml of chloramphenicol at 37 °C and 200 rpm for 18 h. The preculture was then transferred to 1 l of LB media until the A600 reached 0.6 to 0.8. IPTG was added to produce a final concentration of 0.4 mM, and expression was continued at 24 °C and 150 rpm for 18 h. Cells were collected using centrifugation, and pellets were resuspended in lysis buffer comprising 50 mM Tris–HCl (pH 7.5), 150 mM NaCl, 5 mM DTT, 1 mM EDTA, and 10% glycerol before sonication was performed. The supernatant was then collected using centrifugation at 100,000*g* and 4 °C for 70 min, after which it was loaded onto GST Accept (nacalai tesque) and equilibrated using the lysis buffer. Subsequently, the column was washed using five column volumes of the lysis buffer and thrombin cleavage buffer comprising 20 mM Tri–HCl (pH 8.4), 150 mM NaCl, 2.5 mM CaCl_2_, and 1 mM DTT, respectively. The GST tag was removed *via* digestion using biotinylated thrombin (Merck) for 18 h at 4 °C. The biotinylated thrombin was removed using Dynabeads M-280 streptavidin (Veritas). The purity of the target proteins was verified using SDS-PAGE.

### Ligand-binding assay

A ligand-binding assay was performed as described previously with minor modifications (72). Briefly, purified recombinant human PPARδ LBD protein was incubated with 100 nM (10 Ci/mmol) [^14^C] DHA in GP buffer (137 mM NaCl, 8.1 mM Na_2_HPO_4_, 2.68 mM KCl, 1.47 mM KH_2_PO_4_, 1% sodium azide, and 0.1% gelatin) overnight at 4 °C with or without various competitors (1 μM), including DHA and GLA-derived fatty acids. Unbound ligands were removed by adsorption to 3% dextran-coated charcoal, and the supernatant was collected. The PPARδ-bound [^14^C] DHA was measured using a scintillation counter (PerkinElmer).

### Docking simulation

A docking simulation was performed using the AutoDock Vina computational program (38) to predict the binding models of human PPARδ LBD protein with GLA-derived fatty acids. The PPARδ LBD segment of the PPARδ X-ray crystallography structure (PDB: 3GWX, 2AWH, 2BAW, and 2B50) was obtained and used as a receptor.

### Fluorescence detection of fatty acid β-oxidation

Fatty acid β-oxidation activity in living cells was detected using FAOBlue (Funakoshi) (43) per the manufacturer’s instructions. Human intestinal organoids pretreated with 500 μM of oleic acid for 48 h were then treated with 1 μM GW1516 (PPARδ agonist) or 30 μM GLA-derived fatty acids together with oleic acid for 24 h. Embedded human intestinal organoids were incubated with 30 μM FAOBlue at 37 °C for 30 min. The fluorescence signal derived from fatty acid β-oxidation activity was detected using fluorescence microscopy (BZ-X800, Keyence).

### Intracellular triglyceride measurements

Intracellular triglyceride was measured as described previously (73). Briefly, human intestinal organoids pretreated with 500 μM of oleic acid for 48 h were then treated with 1 μM GW1516 (PPARδ agonist) or 30 μM GLA-derived fatty acids together with oleic acid for 24 h. Organoids were harvested using Cell Recovery Solution (Corning) from Matrigel. Intracellular triglyceride was extracted using hexane in 2-propanol (3:2, v/v), and its levels were determined using the Triglyceride Assay Kit Quantification (abcam) and normalized to the levels of total cellular protein, per the manufacturer’s instructions.

### Neutral lipid staining

Human intestinal organoids were harvested using Cell Recovery Solution (Corning) from Matrigel. The organoids were treated with a blocking solution (3% bovine serum albumin solution in PBS). They were then fixed and permeabilized using Fixation/Permeabilization Kit (BD Biosciences), after which they were stained with BODIPY 493/503 (Thermo Fischer Scientific), Alexa Fluor 568 Phalloidin (Thermo Fischer Scientific), and DAPI (Dojindo) according to the manufacturers’ instructions. BODIPY, Phalloidin, and DAPI were used to counterstain neutral lipids, F-actin, and DNA, respectively.

### Statistical analysis

All data are presented as the mean ± SEM. Data were analyzed statistically using one-way ANOVA followed by Tukey’s post hoc test or Dunnet’s test. Statistical differences were considered significant at *p* < 0.05.

## Data availability

All data are contained within the article.

## Supporting information

This article contains supporting information.

## Conflict of interest

The authors declare that they have no conflicts of interest related to the content of this article.

## Figures and Tables

**Table.S1:**
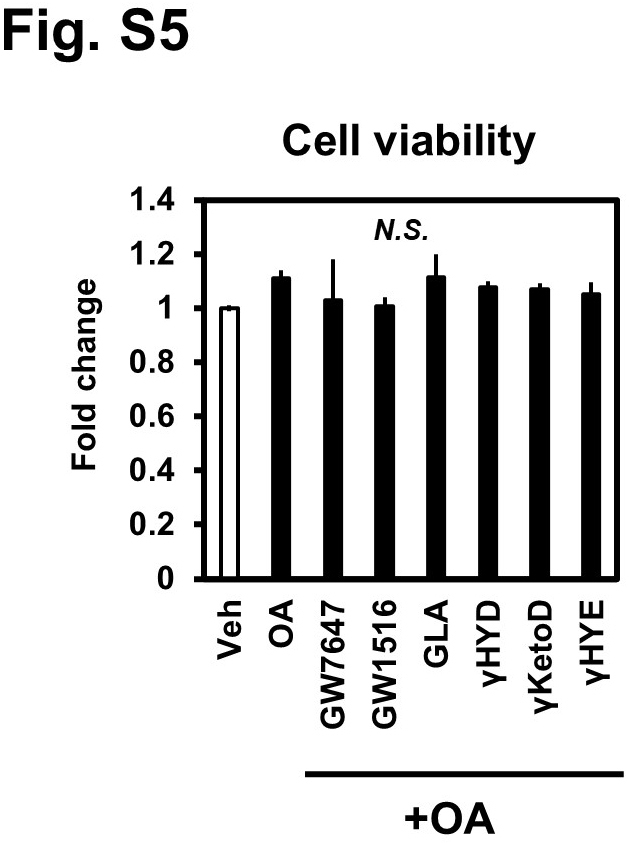


**Table.S2:**